# Histopathological evaluation of latex of Bellaco-Caspi, *Himatanthus sucuuba* (Spruce) Woodson on wound healing effect in BALB/C mice

**DOI:** 10.14202/vetworld.2020.1045-1049

**Published:** 2020-06-10

**Authors:** Lisbeth Lucia Calero-Armijos, Oscar Herrera-Calderon, Jorge Luis Arroyo-Acevedo, Juan Pedro Rojas-Armas, Renán Dilton Hañari-Quispe, Linder Figueroa-Salvador

**Affiliations:** 1 Section of Postgraduate, Universidad Nacional Mayor de San Marcos, Lima 15001, Peru; 2 Department of Pharmacology, Bromatology and Toxicology, Faculty de Pharmacy and Biochemistry, Universidad Nacional Mayor de San Marcos, Lima 15001, Peru; 3 Department of Dynamic Sciences, Faculty of Medicine, Universidad Nacional Mayor de San Marcos, Lima 15001, Peru; 4 Laboratory of Pathology Clinic, Faculty of Veterinary Medicine and Zootechnics, Universidad Nacional del Altiplano, Puno 21001, Peru; 5 School of Medicine, Faculty of Health Sciences, Universidad Peruana de Ciencias Aplicadas, Lima, Peru

**Keywords:** Apocynaceae, BALB/C mice, latex, pathology, regeneration, wound healing

## Abstract

**Background and Aim:**

*Himatanthus sucuuba* (Spruce) Woodson (Apocynaceae) is a medicinal plant known as “Bellaco-Caspi” widely distributed in Loreto, Peru. In the Peruvian traditional medicine, the latex of the plant is used for the treatment of wounds, inflammation, ulcers, and other ailments. This study aims to evaluate the wound healing effect of the latex of *H. sucuuba* in BALB/C albino mice.

**Materials and Methods:**

Thirty BALB/C male mice were used for wound healing study. In the experimental procedures, wound skin incision was performed at 2.0 cm in length until subcutaneous on the paravertebral of each animal. Under locally anesthetized with procaine cream, the treatment was conducted. All the mice were divided into three groups, the control group (A), zinc oxide cream (B), and *H. sucuuba* latex (C). The entire surface of the wound was treated for all the groups, and the treatments were performed daily for 15 days. The experiments were stopped on days 1, 7, and 15, respectively.

**Results:**

The histopathological study of tissues revealed significant changes in wound healing effect in *H. sucuuba* latex compared to the control and B groups. Consequently, the mice treated with latex showed a significant reduction in epithelialization time and collagen formation. Furthermore, the latex showed a dose-dependent significant reduction of inflammation in the first 24 h of treatment.

**Conclusion:**

BALB/C mice treated with the latex of *H. sucuuba* possess a wound healing effect that can scientifically prove the traditional use of the plant as a wound healing agent.

## Introduction

The skin is the body’s first line of immune defense against physical, chemical, or biological aggression from the external environment [1]. Vasoconstriction is the immediate response to physical aggression, which is caused by prostaglandins and thromboxanes; platelets adhere to exposed collagen and the content is released into granules, while tissue factor activates the coagulation cascade and platelets [2]. All body tissues are capable of healing by regeneration or repair mechanisms [3,4]. The process of wound healing is divided into three phases: (a) Inflammation process, (b) proliferation, and (c) tissue remodeling. The inflammation phase includes activation of the innate immune system. During this phase, the differentiation of macrophages, monocytes, lymphocytes, and neutrophils infiltrates the wound site and hemostasis [5]. In addition, pathogens and foreign materials are removed from the wound and prevent damage in a localized area. [6]. The proliferative phase is characterized by angiogenesis, collagen deposition, epithelialization, and wound contraction. Angiogenesis involves the growth of new blood vessels from endothelial cells and these are presented in the tissue remodeling phase. Fibroblasts activate collagen and fibronectin to form a new extracellular matrix in fibroplasia and granulation tissue formation [7].

Plants have an important history in wound healing, as they contain a wide variety of botanical compounds [8]. Medicines derived from plants have been the first line of defense to maintain health and fight diseases [9]. These natural agents provide tissue healing and renewal through multiple mechanisms [10]. Members of the Apocynaceae family constitute an important source of pharmacological discoveries, as they are used for centuries in ethnic and traditional medicine. *Himatanthus sucuuba* (Spruce) Woodson is a shrub plant belonging to the Apocynaceae family, which grows in the Amazon regions of Brazil, Peru, and Colombia, which is known as “janaguba,” “sucuba,” “sucuuba,” and “Bellaco-Caspi” [11]. It is used in common medicine as a vermifuge, antitumor, antifungal, analgesic, anti-ulcer, and anti-arthritic [12]. The practice of traditional medicine has stayed in the majority of the Amazon population and *H. sucuuba* is claimed and commonly used for wound healing purposes, which require scientific validation in Peru.

This study aims to evaluate the wound healing effect of the latex of *H. sucuuba* on wounds induced in BALB/C mice.

## Materials and Methods

### Ethical approval

The Ethical Committee at the Faculty of Medicine of the Universidad Nacional Mayor de San Marcos (UNMSM) (Project N° 0206-FFB-UPG-2018) reviewed and approved this study.

### Study period and Plant material

Leaves of *H. sucuuba* (Spruce) Woodson were collected in Iquitos, Department of Loreto, Peru, in December 2018. In the Herbarium of the UNMSM (Lima, Peru), a voucher was deposited with N° 010-USM-2019.

The latex used in this study acquired a break of *H. sucuuba* stems, which were procured from local farmyard areas of Iquitos. The latex was collected in the morning with a break of stems into a sterile bottle and was stored until further use at 8°C.

### Phytochemical analysis

For the phytochemical screening of *H. sucuuba* latex, different types of reagents were used; the identification results were presented as positive and negative. The amount of sample taken was 1 mL of latex with five drops of each reagent [13].

### Animals

In this study, 30 male mice (*Mus musculus*: BALB/C) aged 12 weeks old and weighing 30-40 g from the Bioterio of the National Institute of Health (INS) were used. All the mice were maintained individually in cages at the Laboratory of Pharmacology, Faculty of Medicine, UNMSM. Pelletized food (Ratonina^®^) and water were given to *ad libitum* for 2 weeks, under controlled conditions of temperature (24°C), relative humidity (70%), and with a photoperiod of 12 h of light cycle and 12 h of darkness. The research was done in the Bioterio of the Faculty of Medicine in the UNMSM (Lima, Peru).

### Experimental design

Thirty adult mice were divided into three groups. The animal’s skin wound was done by means of surgical procedures as explained by Salim *et al*. [14]. The mice were locally anesthetized by topical cream containing lidocaine 25 mg and procaine 25 mg. The control group (A) had no treatment, the standard group (B) applied a commercial cream containing zinc oxide and the latex group (*H. sucuuba* latex) (C). The study was experimental and the dorsal area of the mouse was shaved with a Depile^®^ cream where a transverse incision was made with an N° 20 scalpel on the dorsal-cervical part of 7 mm in length. The effect of *H. sucuuba* latex was observed in the wound area, and the presence of inflammation and re-epithelialization was pointed as markers of wound healing [15]. The treatments were applied once a day with a swab in sufficient quantity to cover the incision. Furthermore, the respective euthanasia was performed on days 2, 7, and 15, using an intraperitoneal dose of sodium pentobarbital [16].

### Histopathological analysis

Through surgical precision, a 1 cm×1 cm tissue sample was taken from the dorsocervical area after the euthanasia. The fixation of the sample was performed with 10% neutral formalin buffer, processed by the classical paraffin inclusion technique. The samples were cut to a thickness of 5–8 μm, staining with hematoxylin-eosin. Histopathological observations were made by light microscopy at two magnifications: 10× and 40×. The captured images were allowed to evaluate the re-epithelialization, presence of fibroblasts, granulation tissue, and inflammatory tissue response. For the diagnosis of the healing study, the qualification criteria proposed by Nguyen *et al*. were used as a reference [17].

### Statistical analysis

The data analyses were expressed by the IBM SPSS Statistics 20.0 (IBM Corp., NY, USA). For the histopathology analysis, the Mann–Whitney U-test was used. p<0.01 was considered statistically significant, which is equivalent to a confidence level of 95%.

## Results

Table-1 shows the phytochemical analysis of *H. sucuuba* latex. The presence of each metabolite was considered as positive or negative by a change of color or any precipitation. More so, free amino groups, phenolic compounds, alkaloids, flavonoids, quinones, saponins, terpenes, and steroids were confirmed.

**Table-1:** Phytochemical constituents of *Himatanthus sucuuba* latex (Spruce ex Müll. Arg.) Woodson.

Test	Results	Phytochemicals
Ninhydrin	+	Free amino groups
Ferric chloride	+	Phenolic compounds
Dragendorff’s	+	Alkaloids
Mayer	+	Alkaloids
Hager	+	Alkaloids
Shinoda	−	Flavonoids
Bornträger	+	Quinones
Foam test	+	Saponins
Liebermann–Burchard	+	Terpenes and steroids

+=Positive, −=Negative

Table-2 shows the qualification of the histopathological index of the wound on days 1, 7, and 15; for the negative control, the positive control (commercial cream), and *H. sucuuba* latex.

**Table-2:** Qualitative analysis of the histological evaluation on wound healing effect of *Himatanthus sucuuba* latex.

Treatment		Results
Day 1	Negative control	Inflammation: Severe Reepithelialization: Mild Granulation tissue: Thin
	Positive control (commercial cream)	Inflammation: Moderate Reepithelialization: Mild Granulation tissue: Thin
	*Himatanthus sucuuba* latex	Inflammation: Mild Reepithelialization: Moderate Granulation tissue: Moderate
Day 7	Negative control	Inflammation: Severe Reepithelialization: Mild Granulation tissue: Thin
	Positive control (commercial cream)	Inflammation: Mild Reepithelialization: Moderate Granulation tissue: Moderate
	*Himatanthus sucuuba* latex	Inflammation: Mild Reepithelialization: Complete Granulation tissue: Moderate
Day 15	Negative control	Inflammation: Moderate Reepithelialization: Moderate Granulation tissue: Thin
	Positive control (commercial cream)	Inflammation: Mild Reepithelialization: Moderate Granulation tissue: Moderate
	*Himatanthus sucuuba* latex	Inflammation: Mild Reepithelialization: Complete Granulation tissue: Abundant

The latex of *H. sucuuba* reduced the time required for epithelialization of the excised wound in mice. A significant (p<0.001) reduction of epithelialization time was observed in mice treated with 100% (w/w) latex, as compared to the negative control group (Table-2).

New vessel formation with scattered inflammatory cells and epithelialization was observed in latex treated mice in the histopathological evaluation of excised skin for 15 days. In fact, when the latex group is compared to the sections from the positive control group (commercial drug), the latex treated mice exhibited a higher number of collagen fibers and fibroblasts. Excised skin of the control group showed an incomplete epithelialization and high grade of inflammation, large ulcers in tissue matrix indicating incomplete wound healing at the end of the treatment.

**Figure-1:**
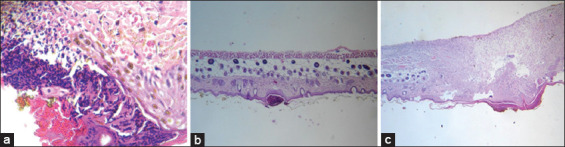
Healing process of the control group with hematoxylin and eosin staining (H and E stain, 40×). (a) Day 1: Loss of epithelium with bleeding and fibrinoid exudate, dense infiltrate of polymorphonuclear leukocytes at the level of the dermoepidermal junction. (b) Day 7: Inflammatory reaction filtered by polymorphonuclear leukocytes and eosinophils, which spread from the superficial dermis to the deepest dermis. (c) Day 15: A large inflammatory reaction to polymorphonuclear leukocytes and eosinophils, which spread from the superficial dermis to the deepest dermis.

**Figure-2:**
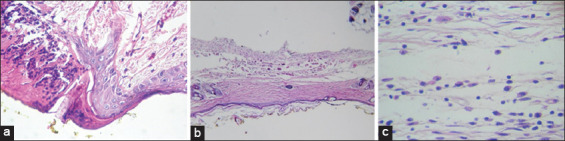
Healing process of the positive control group (cream) with hematoxylin and eosin staining (H and E stain, 40×). (a) Day 1: Inflammatory reaction to polymorphonuclear leukocytes, slightly less intense, but also with injury. (b) Day 7: The treated skin appears normal; absence of annexes (hairs and sebaceous glands), which would be atrophy due to the injury performed. (c) Day 15: Preserved skin, decreased skin attachments (hair and glands), and an inflammatory infiltrate of mononuclear cells, such as lymphocytes and macrophages, indicating less intense, chronic inflammation.

**Figure-3:**
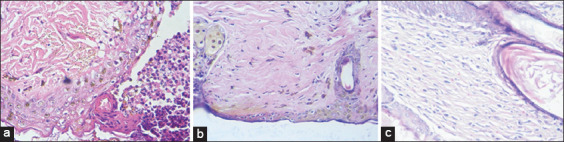
Healing process of the *Himatanthus sucuuba* latex group (H and E stain, 40×). (a) Day 1: Dense inflammatory reaction, but the epithelium is better preserved, two rows of keratinocytes are observed below the superficial infiltrate. Less severe inflammation in the deep dermis. (b) Day 7: An area with fewer adnexa (hairs and sebaceous glands) than in the rest of the skin, and a little more collagen (fibers or thicker). The treated skin appears normal. (c) Day 15: The skin is within normal limits. There is no scar, which would be a fibrous area, with a decrease in skin attachments.

Figures-1-3 show that the latex has a good healing process with very significant differences in the different groups; evidence-based treatment of mild inflammation, complete re-epithelialization, and abundant granulation tissue compared to the positive control (commercial cream) reflecting mild inflammation, mild re-epithelialization, and moderate granulation tissue.

## Discussion

Peru has a rich tradition of plant-based knowledge on health care with a large number of medicinal plants for wound healing and others. *H. sucuuba* contains several medically active compounds such as plumericin, plumericin C, isoplumericin, both bioactive spirolactone iridoids, and four known pentacyclic triterpenes: Lupeol acetate, lupeol cinnamate, lupeol b-phenyl propionate, and a-amyrin cinnamate, which have been isolated from the bark and latex [18]. A study has shown that the bark has a greater antifungal effect than a control drug (nystatin), this action has been attributed to the triterpenic esters found in the bark [19].

*H. sucuuba* is the most studied species, with a record of the chemical composition of the latex, bark, leaves, and roots. The identification of plumericin attributed as a potent new inhibitor of the NF-kB ­pathway provides a solid scientific rationale for the traditional use of extracts of *H. sucuuba* as herbal medicines for the treatment of inflammatory diseases [20]. As shown in Table-1, terpenoids were determined and are considered promising anticancer drugs due to their diverse pharmacological activities, including antiangiogenic, anti-inflammatory, antioxidant effects, and the ability to increase cell differentiation [21].

Latex is used in skin conditions, such as antipyretic, healing of ulcers or wounds on the skin and gastric ulcers, against low back pain, in gastritis, against hernias, herpes, in inflammations of the uterus, malaria, rheumatism, tuberculosis, and tumors [22]. The treated skin with latex appears normal, fairly preserved skin, while the control group decreased its skin attachments (hair and glands), and an infiltrate of mononuclear cells, such as lymphocytes and macrophages, which indicate less intensity and damage in the chronic inflammation. de Miranda *et al*. [23] determined the hexane fraction of *H. sucuuba* inhibited in the edema formation by 35.9% at a dose of 200 mg/kg (p.o.). These results indicate a greater advantage in wound healing of the latex in relation to the positive control.

In addition, the proliferative phase is characterized by angiogenesis, collagen deposition, epithelialization, and wound contraction. Angiogenesis involves the growth of new blood vessels from endothelial cells. Fibroblasts activate collagen and fibronectin to form a new extracellular matrix in fibroplasia and granulation tissue formation [24]. In another report, latex of *H. sucuuba* increased tumor nuclear factor-α, nitric oxide, and decreased transforming growth factor-β production in macrophages which could be involved in its wound healing mechanism [25]. More so, macrophages are the critical key in the wound healing process and provide a useful therapeutic target for wound healing disorders in aging and diabetes, or excessive wound healing in fibrosis [26,27].

There are no toxic, abortive, or birth defects reported in latex of *H. sucuuba*. However, it is necessary to establish its toxic dose or concentrations in the future.

## Conclusion

This study has shown that *H. sucuuba* latex has properties that promote a wound healing and anti-inflammatory effect on BALB/C mice; therefore, further research on latex derivatives could lead to its application as a treatment for post-surgical wounds.

## Authors’ Contributions

LLC and OH conceived the study designed. JLA and LLC performed the experiment. OH and RDH analyzed the data. LF, JPR, and OH drafted and revised the manuscript. All authors read and approved the final manuscript.
